# Twins—An Interesting Case

**Published:** 1887-11

**Authors:** 


### TWINS—AN INTERESTING CASE.

One morning about the last of April, 18—, a handsome blonde female, small in stature, but perfect in form and faultlessly dressed, came into my office for advice, when the following conversation took place:

"Your name and place of residence, please?"

"It matters not who I am, or where I reside. Doctor, I mean business, and hope you will render me that assistance I demand of you, and that at once. This I must have, or I will take my life."

"What is the trouble, madam?"

"I am in the family way."

"How many months?"

"Six."

"Then you have felt your child?"

"Yes."

"If this be so, my good woman, I regret to say you have come to the wrong shop. If you had approached me only a few weeks after the cessation of your monthly sickness, I would have been justified in making effort to re-establish it, for at that early day there is no positive evidence of pregnancy, no sign upon which a physician can rely with certainty. Now, that you have already felt the movements of your child, as you confess, it would be criminal in me to bring on labor, or be in any way accessory to it. If my sympathy and advice can avail anything, they are at your service."

"Thanks. These would be comforting to one less determined than I am. Can't you give me some medicine that will bring me all right?"

"No, madam; not at this stage of your pregnancy."

"Well, then, there is one thing certain, I am determined to get rid of this baby in some way—honestly if I can, desperately if I must, for I would rather die by my own hands than bring disgrace upon my family and children."

"In your anxiety to rid yourself of this child, I presume you have taken medicines to effect it, have you not?"

"Yes, almost without number. Everything known or suggested has been tried."

"What have you taken?"

"I have used two pounds of chloroform in forty-eight hours, and six ounces of ergot in as many hours, besides quantities of balsam-apple, gossypium, turpentine, hot water by injection, and purged and starved myself for weeks, besides resorting to the most violent methods for relief. I have repeatedly jumped or let myself fall from my veranda; I have gone to my kitchen, taken the rolling-pin and beaten my stomach until tender. I have gone to my cellar, laid down on my back and rolled a full barrel of unslacked lime up and down my belly. I have leaned over with my full weight upon my piazza railing time and again, swinging to and fro like a sack. I have ran full force against my wood-pile. I have—"

"My God! my dear woman, stop; that is enough."

"No, this is not all. Will you hear me through?"

"Yes, madam; proceed."

"I have lived in a room saturated with turpentine and other vile odors; drank laudanum, eaten opium and taken morphine with the desperate hope of killing myself. I have gone to th country, mounted the hardest trotting horses and ridden them at a mad gallop over the roughest places. O! how I have struggled to hide my shame."

"Well, madam, my advice to you now is to get out of the city at once, or hide in the humble cabin of some obscure midwife until the show is over, then spirit away your offspring, but by no means destroy it. To carry this out successfully you should impart you secret to no one but the colored nurse—not even to me, for I will not be privy to your crime."

"If an accident should happen to me, doctor, as flooding or convulsions, would you then come to me if sent for?"

"Yes, madam; of course, but not until then."

"Can I trust you further with my history and its secrets?"

"Yes, madam; what takes place in a doctor's office is as sacred as the touch of holy bread; so make yourself easy. I shall not betray you."

About two months after this office interview, and when the little woman had almost passed out of mind, I was called in great haste to see her. I found her exceedingly ill with acute peritoneal inflammation of most violent type, limited seemingly to the left side and in that portion of the peritoneum covering the uterus itself.

Sufice it to say, an abcess formed, discharged itself through the rectum, reformed and again emptied itself in like manner. She remained in bed from May 8 to September 20, a period of five months, and was discharged, as my records show, cured; she has never had a day's sickness of any kind since then, and now walks the streets of this city a beautiful specimen of womanhood. I am still her family physician, to whom she confesses all her physical ills and domestic troubles, but has never alluded to this matter of abortion, and her extreme illness following it, from that day to this, now nearly five years past.

During her convalescence, she gave me the following history: "I am a widow with two children, and 23 years old; whilst visiting friends in a distant State, I formed the acquaintance of a commercial traveler, also widowed. Soon we began to love each other madly. Indiscretion followed under promise of marriage at an early day. I returned home to make preparations for our nuptials. Within a week or so news reached me of his death. I was sad and mourned the loss of my lover as honestly and truly as a woman could, even going into weeds. I soon discovered that I was pregnant, and, like all women in such condition, sought some one in whom I could confide. But my conscience made a coward of me, I left the city secretly and remained away with the intention of concealing my identity as well as my shame. I was troubled, sick, desperate, ready to put into execution any plan by which I could destroy myself, or my offspring, or both. In the midst of my desperation, I sought you with the hope of relief. The sequel has been told you, yet there are some features connected with my case which may interest you, and possibly become a history you can repeat to others alike situated.

About two months after my last visit to your office, a sewing machine agent came to my house to make some repairs on my machine. Whilst talking to him in the hall-way, a pain struck me. I excused myself, went to my room, and before I could reach my bed a baby was born. Catching it, with the after-birth in my hands, I wrapped all together in one of my skirts and hastily thrust the bundle into my wash stand closet. All this happened in about four minutes—perhaps in less time. Returning to my agent in the hall, I assumed the freshness of a daisy and became at once deeply interested in the repairs going on. Suddenly, within five minutes' time, another pain struck me. Again, excusing myself, I hastily returned to my room, reached down standing and caught as before a second baby. This I disposed of in like manner."

"Well, you did not lie down then?"

"No, I had no time to do so. I made for my bed, but was caught half way, so I stood up and performed my work like one bent on destruction. Singular to say, my pains amounted to nothing, simply reminders, as I imagined, of what was to come."

"Do I understand you to say both children were born when you were in an upright position ?"

"Yes; it was fortunate it so happened, for if I had taken my bed, the marks of my struggle would have betrayed me."

"After this, what did you do ?"

"I returned to the hall, feeling quite well, rejoicing meanwhile to find the hated evidence of my shame out of me and out of harm's way. I was unusually amiable, and much more lively than common, and entertained my friend half an hour without the least pain or inconvenience."

"Did you have reason to believe any one suspected what was going on ?"

"No; there was not the least show of suspicion, for I kept up my spirits, drew my corset-strings as tight as I could get them and kept the frolic of my *accouchement* to myself—even in the presence of my children, who all the while went in and out the house at will. I made no effort to keep out of company, but rather sought companionship—particularly those with whom I was most intimate, and joined my children in their sports purposely to deceive."

"What were the sexes of the children ?"

"Both boys—dead ! Yes, thank God ! Dead to every living being, and alive only to Him who gave them to me, and to Him I trust they have returned."

"What disposition did you make of the bodies ?"

"My frolic came off about 2 o'clock p. m. I waited patiently until all had retired for the night and asleep; then, creeping out into the darkness with the two illicit waifs under my skirts, I dug a hole about two feet deep—yonder where the shadows fall, and not a thousand feet away—and covered them well with earth and leaves. When morning came, I was up early, and busy amongst my flowers, taking precious care meanwhile that no evidence of my unholy work remained to betray me."

Thus endeth the first chapter.

